# Whispering-Gallery Mode Optoplasmonic Microcavities: From Advanced Single-Molecule Sensors and Microlasers to Applications in Synthetic Biology

**DOI:** 10.1021/acsphotonics.3c01570

**Published:** 2024-02-03

**Authors:** Matthew C. Houghton, Samir Vartabi Kashanian, Thomas L. Derrien, Koji Masuda, Frank Vollmer

**Affiliations:** † Department of Physics and Astronomy, University of Exeter, Exeter Devon EX4 4QL, United Kingdom; ‡ Department of Life Sciences, University of Bath, Bath BA2 7AX, United Kingdom

**Keywords:** enzymes, thermodynamics, whispering-gallery modes, optoplasmonics, plasmonics, optical microcavities

## Abstract

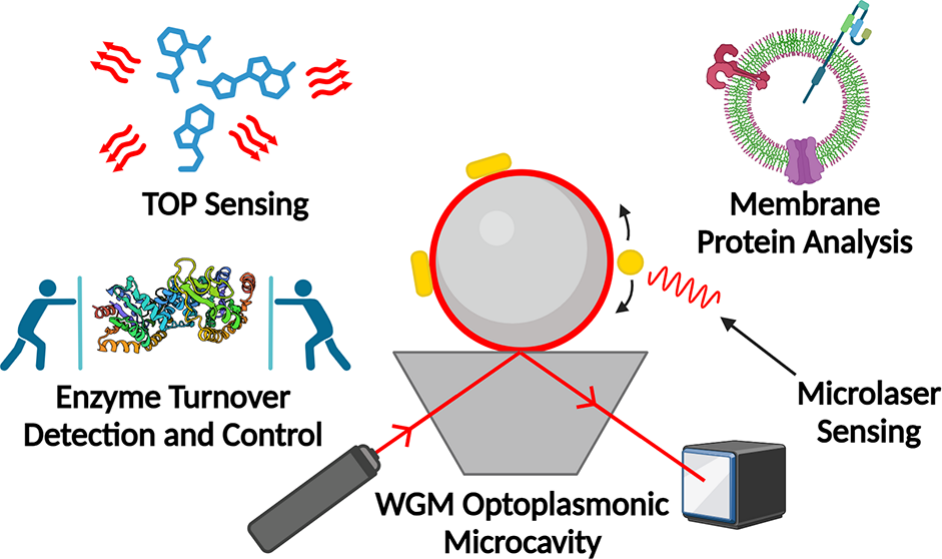

Optical microcavities, specifically, whispering-gallery mode (WGM) microcavities, with their remarkable sensitivity to environmental changes, have been extensively employed as biosensors, enabling the detection of a wide range of biomolecules and nanoparticles. To push the limits of detection down to the most sensitive single-molecule level, plasmonic nanorods are strategically introduced to enhance the evanescent fields of WGM microcavities. This advancement of optoplasmonic WGM sensors allows for the detection of single molecules of a protein, conformational changes, and even atomic ions, marking significant contributions in single-molecule sensing. This Perspective discusses the exciting research prospects in optoplasmonic WGM sensing of single molecules, including the study of enzyme thermodynamics and kinetics, the emergence of thermo-optoplasmonic sensing, the ultrasensitive single-molecule sensing on WGM microlasers, and applications in synthetic biology.

## Introduction

**Figure 1:**
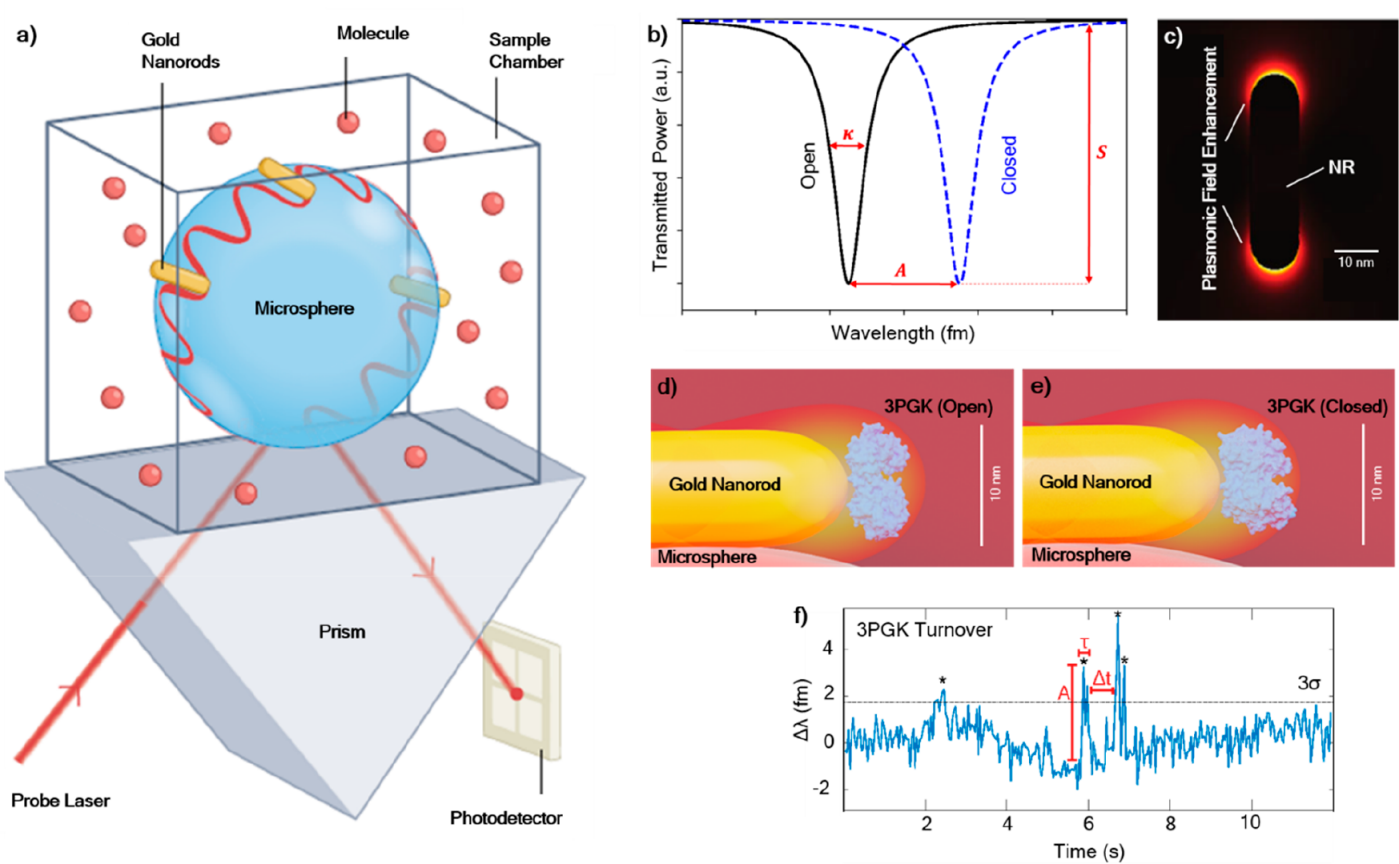
(a) WGM sensor based on a glass microsphere with plasmonic enhancement using gold nanorods. Adapted with permission from Yu et al. 2021^4^ (CC-BY-NC-ND). Copyright 2021 Springer Nature. (b) Wavelength shifts of the transmission spectrum as the polarizability of a molecule bound near the tip of the nanorod changes, in this case, due to an enzyme undergoing an open-to-closed shape (conformational) change. The signal amplitude (*A*) corresponding to the wavelength shift Δλ can be extracted. Other parameters, including the full-width-at-half-maximum (κ) and coupling percentage (*S*) are important for understanding the intensities of WGM involved. (c) Electric field distribution showing field enhancements at the tips of the gold nanorod. Adapted with permission from Subramanian et al. 2021^23^ (CC-BY 4.0). (d) Enzyme turnover at the tip of the gold nanorod, where 3-phosphoglycerate kinase (3PGK) is in the open conformation (2xe6)^24^ and (e) in the closed conformation (2ybe),^25^ when 3PGK has bound substrates and has undergone conformational closure in order to become catalytically competent. (f) Experimental WGM wavelength change (Δλ) depicting several turnover events (*). Several parameters can be extracted: signal amplitude (*A*), signal length (τ), and wait time (Δ*t*). Significance level of 3× the standard deviation (3σ) is marked and of value ≈1.8 fm.

Optical microcavities, essential components in many sensors and lasers, play a pivotal role in various scientific sensing applications. Among them, whispering-gallery mode (WGM) microcavities stand out with their high quality (Q-)factors and small mode volumes.^1,2^ These microcavities confine light by near total-internal reflection along the curved surface of a tiny glass microsphere, typically around 100 μm in diameter.^3,4^ In an aqueous environment, WGM glass microsphere cavities exhibit high-quality optical resonances, boasting high Q-factors, thereby making them exceptionally sensitive to environmental changes.^5^ The remarkable sensitivity of these WGM optical resonances to environmental changes arises from their evanescent fields at the surface of a microsphere that extend to the interaction with the surrounding solution. Microsphere WGM sensors have been widely deployed as biosensors,^6^ enabling the detection of protein monolayers,^7^ biomolecular interactions,^8−10^ bacteria,^11−13^ and nanoparticles.^14−16^ To push the boundary of detection sensitivity to its current highest single-molecule level to enable, for example, the detection of single atomic ions, plasmonic nanorods are strategically placed on the microspheres at the location of a WGM,^17^ as depicted in Figure 1a. These optoplasmonic WGM sensors capitalize on the evanescent field to excite plasmon resonance in plasmonic nanorods aligned with the electromagnetic field, leading to intensity enhancements at the nanorod tips; essentially placing the probing WGM field on the scale of the molecule, where only molecules interacting with the enhanced near-field region at the tips of the nanorods are detectable. These enhancements enable the most sensitive single molecule and atomic ion detection. When a molecule, such as a protein, binds to or near the nanorod tip, it induces a wavelength shift in the optoplasmonic WGM resonance proportional to the excess polarizability of the binding molecule. The resonance wavelength shift serves as a registered detection event, known in the field as the reactive sensing mechanism.^8,18^ Similarly, when a protein that is already bound at the tip of a nanorod changes its shape, for example, because of conformational change, additional detection events are registered on the optoplasmonic WGM sensor; see Figure 1d–f. Single-molecule sensing becomes feasible due to the proportional perturbation of the optical microcavity induced by polarizable molecules like proteins, in tandem with the near-field enhancement of the plasmonic nanorod. Exploring other plasmonic nanostructures, such as nanostars^19^ or nanoparticle dimers,^20^ could potentially offer even higher sensitivity for single-molecule sensing. Additional analyte properties may even be probed; for example, the resonance wavelength shift can indicate the protein’s refractive index in water, and a line width shift can reveal any optical losses due to absorption (and scattering) of light by the protein.^21,22^

These optoplasmonic WGM microcavities open opportunities for investigating challenging biomolecular processes that are difficult to monitor with alternative single-molecule techniques such as fluorescence-based techniques, optical and plasmonic tweezers, and atomic force microscopes (AFM). For instance, optoplasmonic WGM sensors have demonstrated the capability to detect subnanometer-scale enzyme conformational changes in active enzymes such as MalL with microsecond time resolution.^23^ This real-time measurement allows the sensing of the conformational states^26^ of enzymes and measurements of thermodynamic parameters such as activation heat capacity.^23^ Beyond the measurement of enzymatic processes and detection of regular and anomalous DNA hybridization events,^27−30^ optoplasmonic WGM sensing has been demonstrated to be capable of detecting single ions in solution,^31^ as well as single-molecule chemical reactions.^32^ With ongoing advancements, there is potential to enhance sensitivity, readout modalities, and capabilities of optoplasmonic single-molecule sensors, thereby paving the way for diverse new research avenues in single-molecule studies.

This Perspective focuses on some of the most exciting research prospects in optoplasmonic WGM sensing of single biomolecules and is structured as follows:

1) Enzyme Thermodynamics and Kinetics
  a. Sensing Conformational Changes of Active Enzymes and Their Kinetics
  b. Enzyme Thermodynamics and Control of Synthesis
2) Thermo-Optoplasmonic Sensing
3) Synthetic Biology
4) Sensing with Optoplasmonic Microlasers
5) Concluding Remarks

In Enzyme Thermodynamics and Kinetics of this Perspective, how enzymes affect reaction kinetics and the potential of optoplasmonic WGM for studying enzyme conformational changes are discussed. Thermo-Optoplasmonic Sensing introduces thermo-optoplasmonic (TOP) sensing as a novel application of optoplasmonic WGM with implications for measuring forbidden optical transitions in proteins and certain amino acids. Synthetic Biology discusses the application of optoplasmonic sensors in synthetic biology, focusing on their role in controlling the enzymatic synthesis of complex biopolymers and detecting and monitoring membrane proteins with high sensitivity and precision. It highlights the potential for using these sensors to synthesize arbitrary sequence of DNA (*de novo* synthesis) and investigate the translocation of ions and molecules through membrane channels. The combination of optoplasmonic sensors with optical and microfluidic control of enzymatic activity and synthetic cell systems is discussed. In Sensing with Optoplasmonic Microlasers we discuss optoplasmonic microlasers, like silica microspheres doped with rare earth ions, which can provide a cost-effective means for label-free sensing, *in vitro* and *in vivo*, with exceptional sensitivity. These nontunable microlasers require a heterodyne technique to monitor their lasing spectrum, utilizing plasmonic nanoparticles to achieve high single-molecule sensitivity and lowering of measurement noise.

## Enzyme Thermodynamics and Kinetics

### Sensing Conformational Changes of Active Enzymes and Their Kinetics

Enzymes are designed to lower the energy required to reach the transition state (‡) during a reaction, thus reducing the Gibbs’ free energy needed for transition state formation (Δ*G*^‡^-transition state stabilization).^33−35^ However, the kinetics of enzymes is more complex than this. Other factors include destabilizing the substrate ground state (substrate destabilization), which increases the substrate ground-state energy to lower Δ*G*^‡^;^35−37^ substrate binding through induced fit or conformational selection,^38−41^ where energy is released from substrate binding (paradoxical to the former point); and other conformational dynamics.^42^ Observing the conformational dynamics, often critical in order to exclude water and direct reactions in certain directions, is possible via plasmonically enhanced WGM. Optoplasmonic WGM allows for the detection of changes in polarizability of protein molecules within the near-field of plasmonic nanostructures,^43,44^ including conformational changes of enzymes. Recent work by our group has shown the detection of conformational changes of enzymes as small as 23 kDa (*A. aeolicus* adenylate kinase), detecting enzyme radius changes of 0.3 nm. As an enzyme undergoes dynamic changes in shape, the bond angles and distances between atoms change, changing the distribution of electrons in the molecule and, hence, the dipole–dipole interactions between the atoms. This changes the effective molecular polarizability, of which can be calculated using the Thole-modified point dipole model:^45,46^ these changes are detected by optoplasmonic WGM setups as spike-like shifts in the WGM resonant wavelength as an enzyme transiently adopts a conformation upon substrate turnover.^26^ These changes are amplified in optoplasmonic WGM due to the protein moving through a high near-field gradient into areas of potentially higher intensity: a larger number of atoms in a higher field intensity will lead to greater shifts in the WGM resonance wavelength. Other optical techniques can detect single-molecule conformational changes, which include optical tweezer^47^ and single-molecule Förster resonance energy transfer technologies.^48^ However, similar to nanoaperture optical tweezers,^49^ optoplasmonic WGM is label-free, reducing the risk of bulky fluorescent tags or linker molecules attached to AFM tips or microbeads of optical tweezers impacting native enzyme dynamics or activity.^50,51^ Optoplasmonic analysis of enzyme conformation, and with that, kinetics thus far has not been hindered by the close association of the enzymes to the surface of the nanorods.^23,26,52^ However, methods have been developed to utilize His-tags at the C- or N- terminal, present for protein purification, to guard against excessively close interactions with the nanorod surface that may impact enzyme activity.

The use of optoplasmonic WGM for enzyme investigations presents a major assessment of the technique’s capabilities for scientific investigations, providing advantages for biochemical studies by the detection of transient (conformational) states only possible in single-molecule experiments. These detections of conformational change currently allow single-molecule studies of enzyme kinetics- including investigating evidence toward the concept of macromolecular rate theory (MMRT)^53^ through detection of the turnover rate of single enzymes at different temperatures, which allows one to extract (negative) enzyme activation heat capacity ().^23^ This investigation into the MMRT using optoplasmonic WGM represents a major capability of the system for enzyme investigations: it describes a change in conformational dynamics along the reaction coordinate between the enzyme–substrate (ES) and enzyme–transition state (ET^‡^) complexes, only observable in single-molecule experiments capable of detecting transient states.^23,54^ This provides a contribution to a controversial area of enzymology where many insist on dynamics being fundamental for enzyme-mediated catalysis, while others argue not. Optoplasmonic WGM therefore can play its part in the future of this topic with further development of the platform, wider-spread *in-silico* simulation development to understand how optoplasmonics detect these transient states, and a greater availability of the technology for researchers.

### Enzyme Thermodynamics and Control of Synthesis

Optoplasmonic WGM instrumentation can be designed as an investigative tool in enzymology capable of measuring free-energy changes of conformational change (Δ*G*_c_), this being the free energy released as the enzyme collapses from the open, catalytically incapable state to the closed, catalytically competent state. Molecular dynamic simulations have been used to estimate Δ*G*_c_ values in several investigations.^55,56^ However, simulations have their limitations, including approximation of molecular forces involved,^57^ with the ideal scenario being the ability to directly measure Δ*G*_c_. Optoplasmonic WGM may have the potential to measure Δ*G*_c_ by measuring thermodynamic penalties (Δ*G*_p_^*T*^) applied to turnover by the sensors.

As the immobilized enzyme sits in the near-field of the nanoparticles, the enzyme experiences a resistive force when undergoing volume changes associated with conformational change that is dependent on the intensity of the plasmonic hotspot (*I*_WGM_) and near-field enhancement capabilities of the plasmonic nanoparticle. A resistive force works against enzyme movement, as work must be done by the enzyme to move across the near-field gradient created by the excited plasmon resonance:
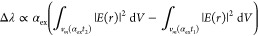
1where Δλ is the magnitude of the wavelength change as the enzyme changes conformation during turnover, α_ex_ is the excess polarizability, *E*(*r*) is the unperturbed electric field, and *v*_*m*_(*t*) denotes the volume occupied by the enzyme in the near-field of the plasmonic nanorod when the enzyme is open (*t*_1_) and closed (*t*_2_).^26,52^ The enzyme must move through the near-field gradient in order to undergo turnover by closing and becoming catalytically competent and then opening again for the next cycle, and these movements are detected as changes in WGM resonant wavelength. By changing |*E*(*r*)| by increasing *I*_WGM_, we increase the resistance against parts of this movement, i.e., for the relevant direction of the open-to-closed conformational transition.

**Figure 2:**
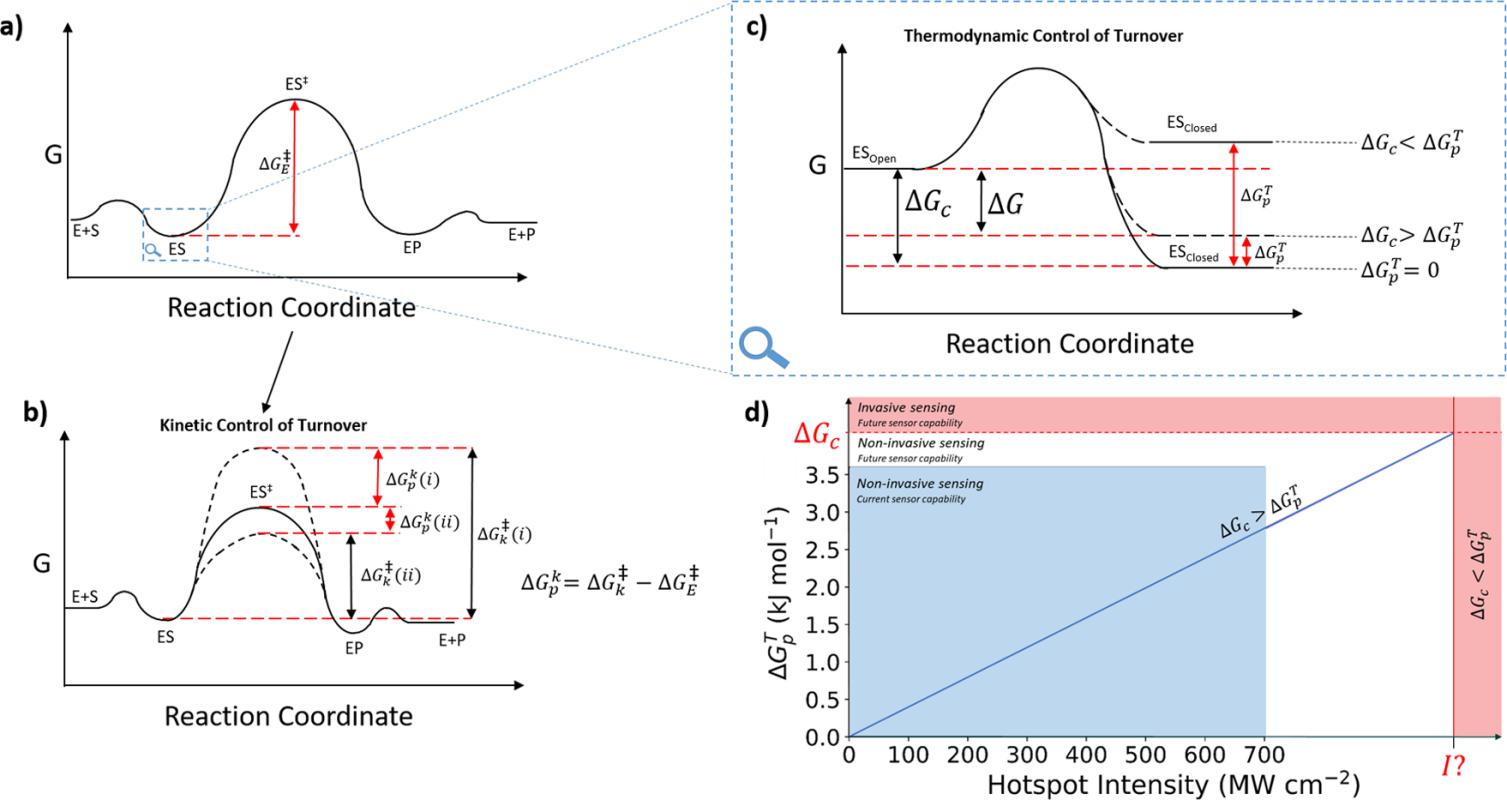
(a) Reaction coordinate of free energy changes during enzyme turnover, according to transition state stabilization theory and ground state destabilization theory. E (enzyme), S (substrate), ES (enzyme–substrate complex), ES^‡^ (enzyme-transition state complex), EP (enzyme–product complex), and P (product). Δ*G*_E_^‡^ is the free energy of activation during the enzyme catalyzed reaction. (b) Kinetic control of enzyme turnover. The manipulation of dipoles within the active site can allow a kinetic penalty (Δ*G*_p_^*k*^), either (*i*) against catalysis, where the penalty increases the energy of ES^‡^ (kinetic penalty), or (*ii*) in favor of catalysis, where the penalty decreases the energy of ES^‡^ (kinetic benefit). (c) Thermodynamic control of enzyme turnover. The application of force to enzyme conformational change provides a thermodynamic penalty (Δ*G*_p_^*T*^) that destabilizes the closed conformation of the enzyme. When Δ*G*_p_^*T*^ is greater than the free energy of closure (Δ*G*_c_), the closed conformation is unfavored and therefore prevents enzyme turnover. (d) Δ*G*_p_^*T*^ vs hotspot intensity graph. As the hotspot intensity increases, the Δ*G*_p_^*T*^ applied to the enzyme increases. As Δ*G*_p_^*T*^ approaches Δ*G*_c_ at intensity *I*?, a currently unknown intensity, the sensor becomes invasive, and near-field gradients of the plasmonic nanorod are strong enough to prevent enzyme movement and ceases turnover.

This resistive force can be quantified as a thermodynamic penalty, Δ*G*_p_^*T*^, and is estimated from the difference in polarization potential of the protein adopting the open and closed conformational states. We propose a method of measuring Δ*G*_c_ by increasing the plasmonic hotspot intensity (*I*_PH_), which combines the *I*_WGM_ and near-field enhancement of the plasmonic nanoparticle, to a level great enough to prevent turnover by preventing enzyme closure or locking the enzyme closed. Practically, this can be achieved by increasing *I*_WGM_ or using different plasmonic nanoparticles with increased near-field enhancement effects such as nanostars or dimeric nanorods. A plot of Δ*G*_p_^*T*^ vs *I*_PH_ will reveal Δ*G*_c_ as the value of Δ*G*_p_^*T*^ at which the enzyme turnover is perturbed (Figure 2d), due to the free energy change of the system now being positive and nonspontaneous (Δ*G*_system_ = Δ*G*_c_ + Δ*G*_p_^*T*^ > 0). By preventing enzyme conformational change and hence turnover, this could demonstrate the potential to control single enzymes by applying optical forces (Figure 2c). This could lead to the development of systems capable of an enzymatic synthesis of single molecules of complex polymers such as DNA with a specific sequence by switching enzymatic activity on/off, dependent on the desired nucleotide, for example. Other advantages to the optoplasmonic WGM system include the potential ability to detect the effects of smaller forces applied to proteins and enzymes than optical tweezers (0.1–100 pN)^58^ and AFM (20 pN to 10 nN).^59^

Control of enzyme activity could take place by two methods: the above method explains a thermodynamic method. However, there is also scope for control via kinetic means. Warshel proposes in several publications^35,60,61^ that the preorganization of the active site allows electrostatic stabilization of the transition state and includes protein charges, permanent dipoles, and induced dipoles. The latter of which could be influenced by high intensities of optoplasmonic WGM: the near-field of plasmonic nanoparticles may be able to induce dipoles or affect protein polarity.^62^ It is known that charge reorganization is a possible form of allostery of protein activity.^63,64^ Therefore, we proposed that high intensities of optoplasmonic WGM could destabilize the dipoles within the active site and hence change Δ*G*^‡^ (Figure 2b).^65^ Likely in a case-by-case manner, this could increase (kinetic benefit) or decrease (kinetic penalty) the rate of reaction, allowing control of enzyme turnover by kinetic means.

## Thermo-Optoplasmonic Sensing

Optoplasmonic WGM has traditionally been used for the detection of single molecules^30^ and even single ions^66^ due to the enhanced sensitivity. These detection methods use the reactive sensing regime, in which polarizability changes occur when particles enter the hotspot of plasmonic nanorods excited by WGMs, producing red (Δλ > 0) or blue (Δλ < 0) permanent, step-like wavelength shifts depending on each molecules polarizability.^8,14,67^ The excess polarizability of biomolecules in water is typically positive, hence, positive wavelength shifts have been observed in optoplasmonic single-molecule studies.^52^ Instead, recent work by our group has shown that different mechanisms of sensing are possible with optoplasmonic WGM sensors. We report a new sensing regime, termed thermo-optoplasmonic (TOP) sensing.^21^ In this regime, the absorption of energy by the protein from the WGMs circulating the resonator and subsequent relaxation results in energy release as heat, which slightly heats the surrounding water (on the order of 1 or a few K). This heat changes the local refractive index in the near-field of the plasmonic gold nanorod upon protein binding, resulting in a negative contribution to the wavelength change because the refractive index of water decreases with increasing temperature (d*n*/d*T* = −1.3 × 10^–4^ K^–1^). In doing so, at high *I*_PH_, this negative contribution is great enough to outweigh the polarizability change of the protein and thus switch the Δλ sensor signal from a previously positive to negative magnitude.

A change in sign of Δλ at a threshold intensity, typically 90 MW/cm^2^ at the nanorod tip, is characteristic of TOP sensing. For proteins labeled with small-molecule dyes, this is expected when they have high absorption at the WGM wavelength and low quantum efficiency. However, the high Q-factor of WGMs and nanorods harboring oscillating electrons allows forbidden optical transitions to be accessible. Traditionally, protein molecules do not absorb at 780 nm, which is a popular wavelength for WGM experiments. We report TOP sensing observed on proteins containing tryptophan but not in those without. This demonstrates a forbidden optical transition accessed by electron capture from the plasmonic nanoparticle onto the indole ring of tryptophan in proteins,^67^ increasing their apparent absorption cross-section at 780 nm.^21,22^ TOP sensing poses the possibility of new absorption spectroscopy technologies and methods for measuring forbidden transitions. Future advancements in this field will allow TOP sensing regimes to be characterized at many different wavelengths. It has also enabled better understanding of intensity in WGM experiments through a new model:
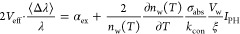
2where the effective mode volume of the WGM based on the local intensity at the nanorod’s hotspot (*V*_eff_), initial wavelength (λ), wavelength shift (Δλ), excess polarizability (α_ex_), absorption cross-section of molecule under test (σ_abs_), refractive index of water (*n*_w_(*T*)) at temperature *T*, water’s thermal conductivity (*k*_con_), effective volume of the heated water (*V*_w_), effective heat transferring length (ξ), and plasmonic hotspot intensity (*I*_PH_) are considered.^21^ This new model characterizes the nature of Δλ accounts for both polarizability changes (reactive sensing) and absorption of WGM energy (TOP effects), unlike previous models, providing a more comprehensive understanding of optoplasmonic WGM sensor behavior.

## Synthetic Biology

On one hand, synthetic biology endeavors to redesign living systems, from genetic codes to cells and organisms, by conferring novel capabilities that exceed what nature provides. Synthetic biologists harness the tools of molecular biology, including enzymes and genetic materials, to meticulously modulate intricate biological processes, yielding the desired outcomes in medicine, manufacturing, agriculture, and the environment. On the other hand, optoplasmonic WGM sensors have achieved the sensitivity capable of the detection of subnanometer conformational changes of active enzymes. The nanosensor system combined with a laser lock-in technique has enabled the real-time tracking of turnover of a single enzyme on the sub-ms time scale without the use of fluorescence label.^23^ As discussed in the previous section, the thermodynamic penalty imposed on an enzyme due to the resistive force exerted by the near-field implies the potential of controlling single enzymes on the nanosensor system. These demonstrations, which highlight the exceptional capabilities of optoplasmonic WGM sensors in monitoring and modulating the molecular dynamics of individual enzymes, unlock opportunities to explore various strategies for controlling enzymatic processes at the single-enzyme level and paving the way for an emerging research area at the interface of WGM biosensing and synthetic biology.

One can consider, for instance, the immense potential of harnessing the high sensitivity of optoplasmonic WGM sensors at the angstrom length scale and their potential micro/nano second time resolution to control enzymatic synthesis in real time. Various strategies, including the following examples, can be integrated with the optoplasmonic WGM sensor platform for the real-time control of single-molecule enzyme activity. An initial step to realize enzyme control using the WGM platform would require temperature control of single enzymes. Enzymatic activities can vary by over 10-fold across a temperature range from below 10 °C to their optimal temperatures, which typically occur around 37 °C for mesophiles. Laser heating is a simple method requiring only an external laser tuned to the water absorption peak. For a small volume (a few nL), it is possible to heat or cool by a few tens of °C within a second.^68^ However, the exceptional sensitivity of the optoplasmonic WGM sensor, with a sensitivity of 10 pm/K in the case of heating the glass microsphere cavity, makes it susceptible to temperature drift. Therefore, it may be required to consider mitigation strategies to ensure that the sensor remains within its operational range depending on the platform. Plasmonic heating is another heating method that is highly localized within the region extending only ∼100 nm from the nanoparticle surface, enhancing both time response and thermal stability. At this length scale, plasmonic heating of a nanoparticle occurs instantaneously, with temperature equilibrating in less than 100 ns in water.^69^ For microsecond heating pulses, the average thermal drift of the optoplasmonic WGM sensor is expected to be minimal. Potentially, enzyme activity can be more directly controlled by means of optical resistive forces as discussed previously.^70^ The steep field gradient of the plasmonic near-field on the surface of nanoparticle can generate a potent force capable of modulating the conformational dynamics of an enzyme, hence regulating the enzyme’s activities without employing an external laser. In conjunction with the optical methods described above, segmented flow microfluidics offers an effective method where minuscule nanolitre droplets of aqueous reaction mix are isolated with an oil phase, forming encapsulated microreactors.^71^ This microfluidic approach, ideally in conjunction with regulating enzyme activity by one of the approaches discussed before, could grant precise temporal control over the chemical environment surrounding the optoplasmonic WGM sensor volume and the enzymatic reaction.^71^ Essential reaction parameters, including the composition of the reaction mixture, pH levels, and residence time, could be meticulously controlled. This would lead to the possibility of on-demand delivery of desired reagents and substrates for controlled enzymatic reactions, substrate molecule by substrate molecule.

**Figure 3:**
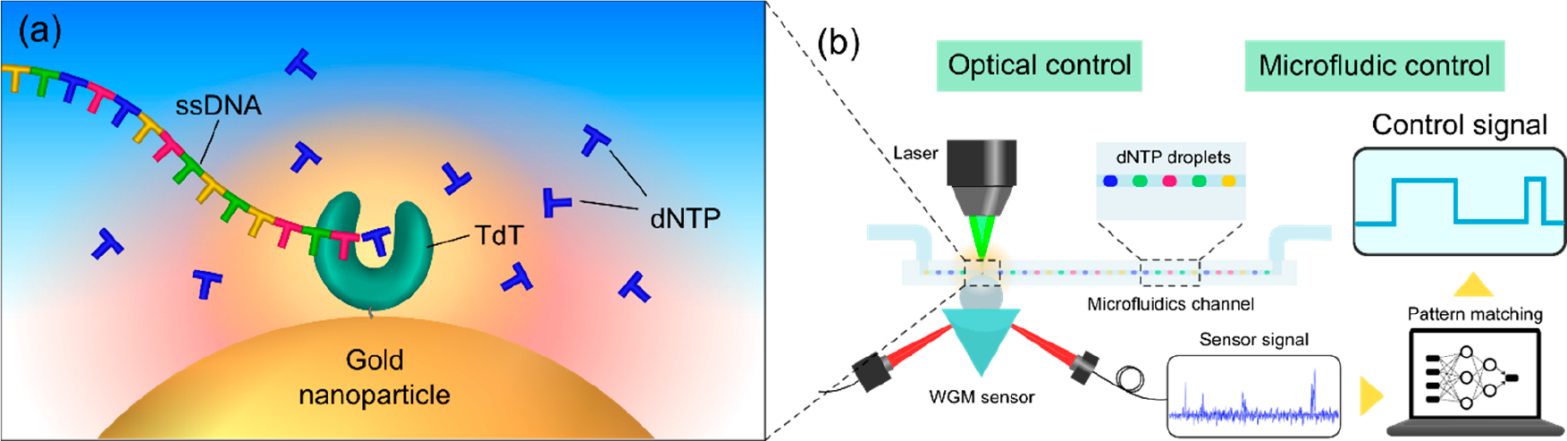
*De novo* DNA synthesis based on an optoplasmonic sensor could potentially address the challenge of synthesis of long, error-free DNA strands. (a) The sensor monitors conformational changes of a surface-immobilized TdT enzyme and the interaction between TdT, single-stranded DNA (ssDNA) and nucleotides (dNPTs) near the gold nanoparticle surface. (b) Precise synthesis of sequence-defined ssDNA can be achieved by real-time control of enzyme activity via plasmonic heating/optical force (optical control) and the on-demand delivery of desired reactants using droplet microfluidics (microfluidic control).

Such combined capabilities can address current challenges in synthetic biology, including a critical need for advancing methods to achieve *de novo* DNA synthesis.^72−74^ Recently, Terminal deoxynucleotidyl Transferase (TdT) has emerged as a promising candidate to achieve this, as TdT can nonspecifically add any nucleotide to a growing single-stranded DNA.^75,76^ It is conceivable that by monitoring the activity of the individual TdT enzyme on an optoplasmonic WGM sensor and providing real-time feedback signals to actuate one or a combination of the control mechanisms, for instance, through temperature control, described above for regulating the TdT enzyme activity, one can facilitate the precise incorporation of specific nucleotides into the growing DNA strand. This process allows for the synthesis of potentially any desired DNA sequence with a high degree of accuracy. Figure 3 describes the concept of *de novo* DNA synthesis based on the optoplasmonic WGM sensor platform where the activity of a single TdT enzyme is monitored and controlled in real time via the optical and microfluidics feedback to facilitate sequence-defined synthesis of single stranded DNA. Once synthesized, the DNA sequence can be scaled up by follow-on PCR reaction(s).

A summary of the potential methods for controlling enzymatic activity using WGMs is presented in Table 1. While contemporary synthetic biology pertains to the engineering of macroscopic biological systems, such as cells, the optoplasmonic WGM sensing platform promises synthetic biologists a nanoscale instrument to investigate, design and precisely control microscopic biological systems, operating at the level of single molecules and with exceptional temporal accuracy. This unique capability allows researchers to explore the uncharted realm of individual molecules, potentially uncovering novel design principles and innovative strategies for biomanufacturing of functional biomolecules and proteins. In DNA engineering, the single-molecule control of enzymes could lay the foundation for new methods of DNA nanomanufacturing (e.g., DNA origami)^77^ and DNA-based information storage.^78^

**Table 1:** Summary of Various Strategies for Controlling Enzymatic Activities on the WGM Platform

modality	method	description
optical	laser heating	Heating surrounding water by a few 10s of °C in less than a second. Simple to implement.
	plasmonic heating	Rapid and localized heating of plasmonic nanoparticle in subms scale with minimal effects on WGM sensing.
	optoplasmonic force	Direct control of enzyme motion by near-field gradient force. No extra laser required.
microfluidic	segmented flow	On-demand delivery of desired substrate and reagents to the sensing volume.

**Figure 4:**
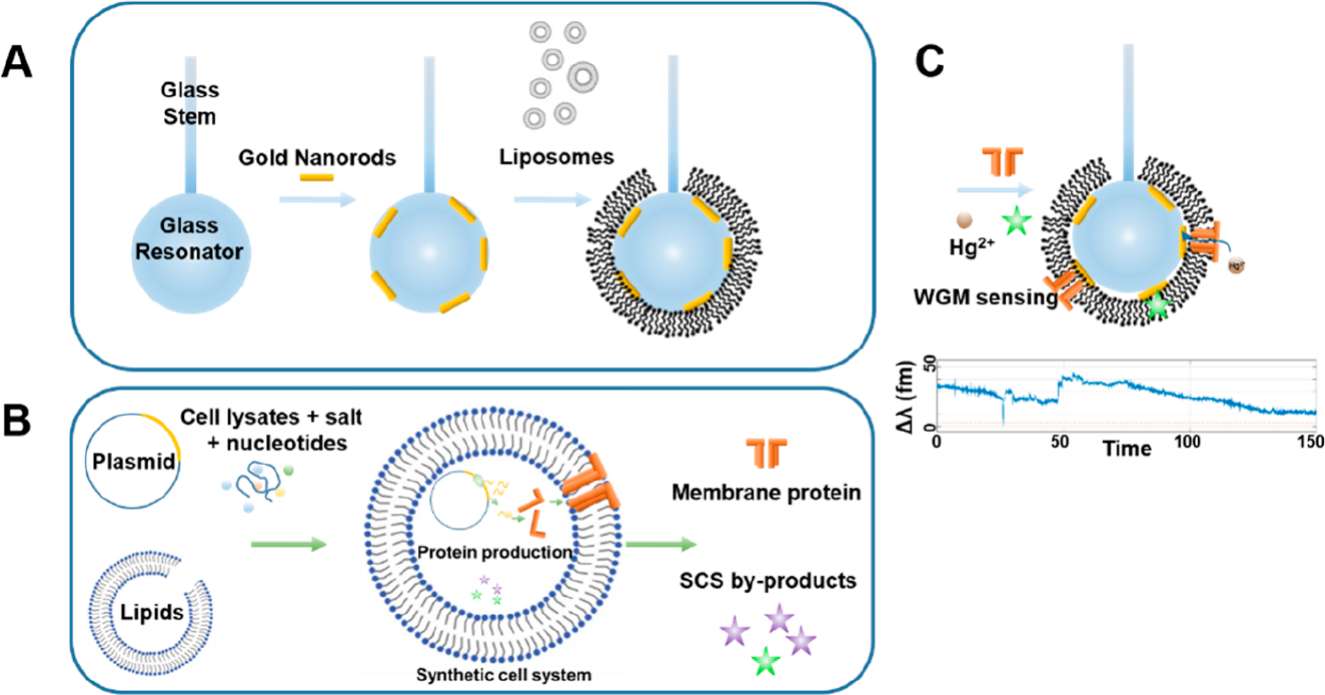
Merging synthetic biology and WGM technologies. (a) Coating WGM resonators with lipid bilayers to enable the detection of membrane proteins and molecules that associate with the membrane. A nanoplasmonic WGM sensor is first formed by depositing gold nanorods onto the surface of the resonators; Liposomes incubated with a glass resonator will form a supported lipid bilayer to create a platform for membrane sensing. (b) Using synthetic cell systems for protein production or the production of specific metabolites which can then be incorporated into a (c) WGM detection scheme. Various sensing schemes can be envisioned with this platform such as detecting SCS produced membrane protein insertion, such as α-hemolysin, or the byproducts formed from the SCS transcription-translation process. The translocation of WGM detectable ions, such as mercury could then be probed across the membrane and show up as steps in the WGM spectra (c-bottom trace), creating a new sensor for the detection of translocating ions.

One emergent aspect of synthetic biology is the construction of synthetic cell systems (SCS), engineered particles that recapitulate specific biological functions of cells, especially protein production by DNA transcription and RNA translation, for applications such as drug delivery of bioremediation.^79−82^ SCS typically employ lipid bilayers to compartmentalize the genetic material and macromolecules necessary for protein production, and the development of active membranes remains a challenge. By coating an optoplasmonic WGM microsensor with a membrane layer (Figure 4a) and probing the functional aspect of these membranes with high resolution and at the single molecule level, for example by detecting the insertion of SCS produced membrane proteins and their dynamics, can lead to new developments to advance the field of synthetic biology (Figure 4b). A coating of the membrane on the approximately 100 um microsphere sensor made of silica could be achieved by vesicle fusion (Figure 4a) or by Langmuir–Blodgett deposition directly onto a prefabricated resonator. As the microsphere resonator is formed by melting an optical fiber, a glass stem remains, which could potentially be used to transfer into several other solutions for further membrane modification (Figure 4a). SCS in the presence of WGM sensors could be used to probe the functionality of SCS expressed membrane proteins, such as the pore forming protein α-hemolysin (α-HL), which functions as a nonspecific passive membrane channel. Combining the capability of WGM sensors to detect single ions (Zn^2+^ and Hg^2+^)^31^ and SCS shown to express α-HL^83^ would open up the possibility of detecting single ion translocation events through membrane pores (Figure 4c).

Single-molecule sensitivity can be achieved only when the analytes interact with the plasmonic nanoparticles coupled to the sensor. Therefore, chemistry to ensure that the proteins of interest interact with the plasmonic nanoparticles once inserted into the membrane must be developed. Furthermore, signals that originate from the membrane and its fluctuations must be discerned from those that originate from the membrane proteins. various methodologies have been developed^52^ to covalently conjugate proteins to the nanoparticles, such as gold–thiol bonding using native cysteine present or through the insertion of cysteine mutants via genetic engineering.^9,23^ Other methods to conjugate proteins to the nanoparticles, such as “click” chemistry^84^ or EDC-NHS^85^ coupling, have also been demonstrated.

Single-molecule detection offers countless advantages to bulk detection, as mentioned above. Synthetic biology offers another advantage: expressing proteins starting from DNA such as difficult to purify membrane proteins. The combination of optoplasmonic WGM membrane sensors combined with SCS could enable single-molecule experiments with membrane proteins such as olfactory receptors or ion channels in neuroscience. Alpha-hemolysin could be a well-established test system to show the capabilities of this approach. It may even differentiate signals for small molecules translocating the membrane channels through interactions with the gold nanorod on the optoplasmonic sensor.

Another interesting membrane-associated protein system to study is the *E. coli* Min system, which is responsible for the placement of bacterial cell division machinery and involves collective oscillations of the three proteins MinC, MinD, and MinE.^86^ These oscillations are the result of the bulk movement of proteins onto and away from the bacterial membrane and could thus be detected onto a membrane coated WGM resonator, perhaps from an SCS system expressing these proteins. A WGM based biosensor for the detection of membrane associated proteins could provide essential insight into the factors contributing to pattern formation and oscillation in the Min system, such as lipid composition and affinity. This could be achieved by using WGM coupled SCSs expressing engineered Min proteins to probe the binding effect of altering the sequence of the lipid binding domains^87^ and monitoring the oscillations in vitro.

These approaches illustrate the potential for combining WGM sensors with synthetic biology to create novel solutions for molecular detection and real-time monitoring of biological processes down to the single-molecule level. Further innovations can be imagined by incorporating WGM sensors with microfluidics for the controlled capture and manipulation of SCS. Such advancements could have applications in medical diagnostics, drug development, and fundamental biological research.

## Sensing with Optoplasmonic Microlasers

Microlasers are one of the most powerful label-free sensing platforms for single nanoparticles, viruses, and even multispecies and individual gas molecule detection.^88,89^ WGM microlaser properties and fabrication methods have been extensively studied. Among many methods of fabrication, from quantum dots (QDs) to organic dyes and from microbubbles to microtoroids,^90^ the most robust and cost-effective fabrication method is doping silica microspheres with rare earth ions.^91,92^ The simple level structure and higher gain efficiency of Yb^3+^, which enables laser operation at low-quality factors, make this ion a favorable dopant for sensing applications. In addition, low absorption at the Yb^3+^ emission band (λ_em_ = 1030–1100 nm) in aqueous environment, compared to Er^3+^ (another popular dopant) with λ_em_ = 1530–1570 nm, is an important factor for biosensing applications. Normally the spectral property of a passive cavity is continuously measured by using a scanning laser probe beam. This is commonly called the spectral shift technique applied in a reactive sensing scheme. The cavity resonance and line width strongly depend on the environment. Any change in the refractive index of the cavity and/or the surrounding environment, i.e., temperature fluctuations, can shift the resonance and change the line width. The sensitivity of such system is determined by *Q*/*V*, where *Q* is the Q-factor and *V* is the mode volume of the system. Moreover, in optical resonators, the local thermal fluctuations, which are particularly strong in small optical mode volumes, lead to refractive index noise and result in noise and instability in the resonance frequency. This is known as thermorefractive noise and limits the detection sensitivity of the passive optoplasmonic WGM resonators.

In active cavities on the other hand, the gain material increases the losses in the cavity due to absorption and scattering, and thus a lower Q-factor is expected. However, microlasers could be ultrasensitive above the lasing threshold as the Purcell effect narrows down the cavity line width compared to the corresponding cold or passive resonator, which eventually leads to Q-factor enhancement. Simulation results^93^ show that the line width of a lasing microsphere could be 10^4^-fold narrower than the line width of the corresponding passive resonator. In the case of microlasers based on optoplasmonic WGM, this could lead to single molecule sensing at unprecedented levels of sensitivity.

Since microlasers are not tunable, monitoring their lasing spectrum requires a heterodyne technique. This technique involves introducing plasmonic nanoparticles (NPs) to the microlaser, which changes the resonance properties of the system. Plasmonic gold nanorods (NRs) deposited on the microlaser’s surface result in a near-field enhancement, providing the necessary sensitivity for detecting single biomolecules on optoplasmonic WGM sensors.^31,52^ When a molecule interacts with an NR, the resonance wavelengths of the standing wave modes (SWMs) shift, creating a beatnote frequency that indicates the presence and interaction of individual molecules. The response of these split modes to temperature fluctuations is the same and thus the thermorefractive noise is canceled in the beatnote frequency, which was demonstrated for nonplasmonic microlasers interacting with dielectric nanoparticles.^94^

**Figure 5:**
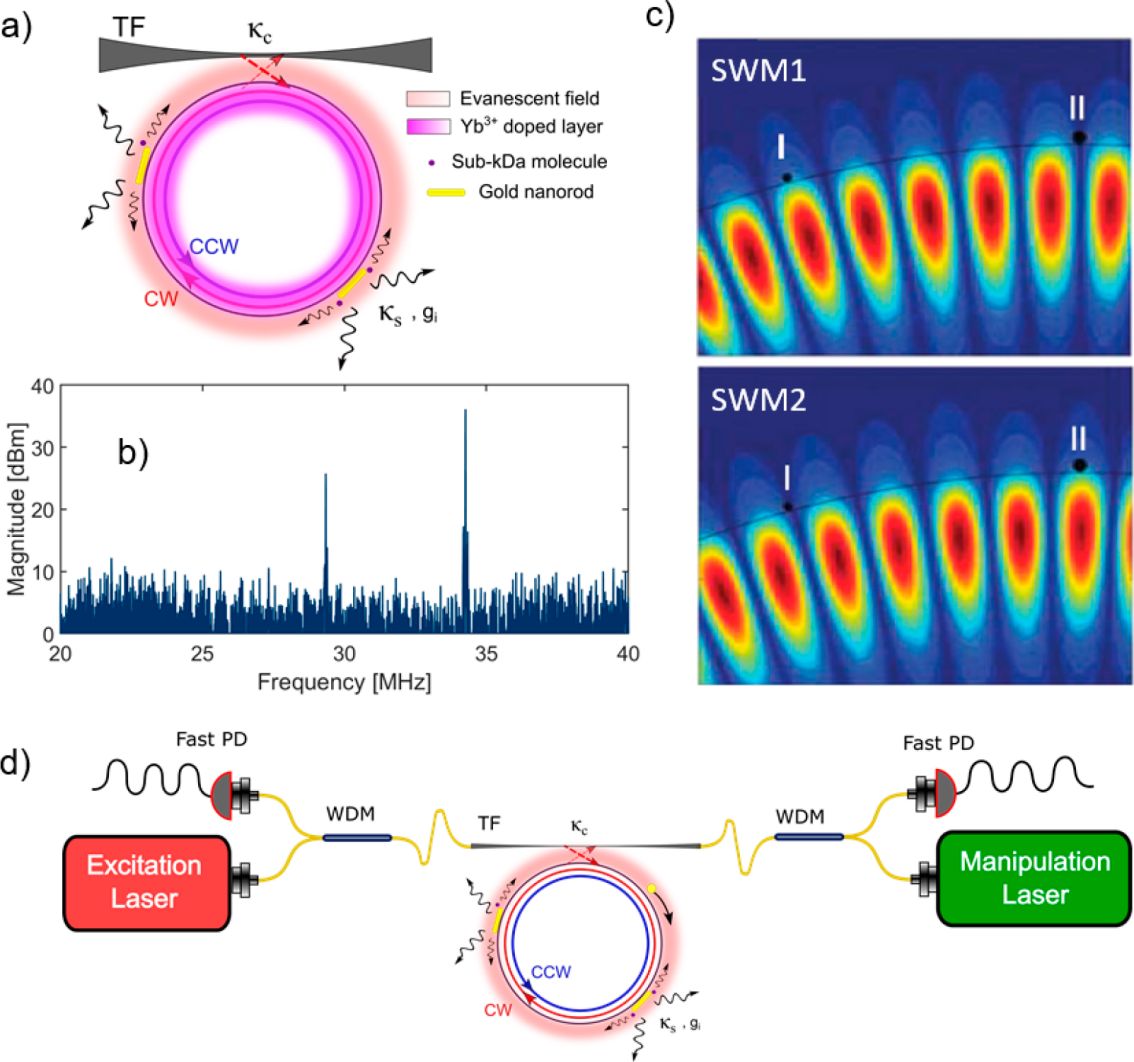
(a) An optoplasmonic WGM microlaser excited by an excitation laser beam through a tapered fiber (TF). The initial excitation is in the CW direction. However, by introducing plasmonic gold nanoparticles, which act as a nanoantenna for single molecule detection, SWMs form with nondegenerate eigenfrequencies. This mode splitting between CW and CCW WGMs is reflected as a beatnote in the lasing output. Small molecules interacting with the gold NRs alter the splitting frequency of the SWMs. (b) is an example of microlaser beatnotes at near 30 and 35 MHz. Two beatnotes represent two independent lasing modes taking place in the microresonator. (c) The field distributions of SWM1 (top) and SWM2 (bottom) when multiple gold NPs are attached to the microcavity. The splitting between the two SWMs is clear as a phase difference. I and II are nanorods bound on the microresonator, interacting with both SWM1 and SWM2. Adapted with permission from Subramanian et al. 2020^97^ (CC-BY 4.0). (d) The schematic of the optical setup required for steering carousel NP on an active WGMR. Bidirectionality of the beatnote detection is required to study the directionality of the lasing near EPs. Dual lasers in the counterpropagating scheme are required to compass the carousel to a desired point and fine position it while maintaining the optical trap.

Consider a WGM resonator with some Rayleigh scatterers, such as plasmonic NPs, deposited on the surface (Figure 5a). Light is initially coupled into the resonator in the clockwise (CW) direction. However, the scattering couples both the CW and counterclockwise (CCW) modes. The presence of the NP Rayleigh scatterers lifts the degeneracy between the CW and CCW modes and forms two nondegenerate standing wave modes (SWMs), which results in a slight detuning between those modes known as mode splitting, analogous to the strong coupling in cavity-QED.^95^ In active optoplasmonic WGM the lasing modes occupy both SWMs simultaneously and the interference between them results in a beating in the lasing intensity at the same frequency as the detuning between the split modes. This naturally provides a self-heterodyne system where the detected microlaser beatnote gives the spectral properties of the resonator. The observed beatnote frequency reflects the detuning between the split modes which depends on the polarizability of the NPs, their interaction strength with the cavity, and their relative spatial phase^96^ (Figure 5c), as well as the interaction with a molecule.

The plasmonic gold nanorods (NRs) on the surface of an optoplasmonic WGM locally enhance the electromagnetic field and provide enough sensitivity to detect single biomolecules. When a molecule interacts with an NR, the SWMs resonance wavelength shifts according to the overlap of that NR with the two SWMs. In other words, the magnitude of the molecular binding induced resonance shift for each SWMs, which is always a red shift (δλ > 0 for biomolecules in water), depends on the local field enhancement at the molecule binding site on the NR delivered by each SWM (Figure 5c). Therefore, by introducing the molecules to the NRs, the beatnote (splitting) frequency shifts either in the negative or positive direction.^97^ In WGM microlasers, these beatnote frequency shifts could be used for the ultrasensitive detection of binding or conformational/state transitions of individual molecules. In passive optoplasmonic WGM, the split modes are often unresolvable, limiting the split-mode technique currently to only nanoparticle detection.^97^ However, in a microlaser it is straightforward to detect and resolve split modes by monitoring the microlaser beatnote frequency and establish optoplasmonic WGM microlasers for ultrasensitive single molecule detection.

The proposed single-molecule beatnote sensing approach would work with immobilized plasmonic NRs. However, NPs can be optically trapped and orbit around a WGM resonator (WGMR) within its evanescent field. This carousel trap has been realized using a passive WGMR in an aqueous environment with polystyrene nanospheres and derived at low optical powers. Individual NPs are attracted by optical gradient forces and orbit due to the forces by circulating momentum flux.^98^ However, using WGM microlasers and beatnote techniques gives a more sensitive signal to the radial position and fluctuations of the carousel NP, due to Brownian motion, and relative spatial phase between NPs in the sensing ring. Moreover, the laser frequency scan approach (which is essential in the passive WGM resonators) is avoided in the beatnote technique, and thus in principle it reports the forces affecting the NP in the carousel trap and position of the NP much faster.

As the carousel orbits around the WGM resonator and radially fluctuates due to Brownian motion, the beatnote signal varies. The carousel reports information about nanoscale activities of the NPs and the forces and interaction between the moving NP and the stationary NRs, by changing the beatnote frequency, magnitude, and line width. There is high demand for the development of a fast data acquisition method that follows the fast dynamics and fluctuations in the beatnote signal. This is achievable by using an RF discriminator that converts the magnitude and frequency of the beatnote to a voltage signal and assures a fast read out and data logging mechanism.

In addition, a carousel NP can be employed to realize an exceptional point (EP) and further to achieve EP enhanced sensing of single particles and molecules. However, for practical reasons, the EP is realized by introducing a few stationary NRs and a finely positioned nanotip. EPs emerge when a non-Hermitian system is tuned such that its eigenmodes and eigenvalues coalesce. EPs have previously been achieved by a single active WGMR and multiple dielectric nanoscatterers.^99^ Introducing few NRs to a WGMR leads to formation of two SWMs and splitting in their corresponding mode resonances (nondegenerate system). By finely adjusting the position of the nanotip relative to other immobilized NRs, the degeneracy in the system can be recovered, and thus the splitting between SWM1 and SWM2 coalesces, and the beatnote frequency drops to zero. The higher order EPs (with 2 or more stationary NRs) could enhance the sensitivity to the perturbations at even the single molecule level. As the nanotip or NP moves around the surface of the resonator, the system transits from a split mode system to EP. The beatnote provides an elegant monitoring tool to extract information about the NP scatterers, which generate and determine the beatnote properties. Any other excess perturbations, such as local optical and mechanical forces on the NPs and their displacements, induce some variations to the beatnote frequencies, magnitudes, and line widths. When these variations are above the noise level in a system, particle/molecule detection is obtained.

Another exciting and ambitious biosensing application with active optoplasmonic WGM microlasers would be *in vivo* and *in vitro* diagnosis. Developing lab-on-chip *in vivo* devices that analyze biological samples is the goal for many biosensor platforms. Here, WGM microlasers can be employed to achieve *in vivo* single molecule detection in ultrasmall volumes.^100^*In vivo* WGM microlasing has been demonstrated by injecting dyes into a living cell as the gain.^101^ It is possible to implant a hybrid active optoplasmonic WGM in an embryonic zebrafish when it is transparent. In this proposed application, a rare earth ion-doped silica WGM microlaser with few embedded NRs is implanted in a zebrafish. A confocal microscope is employed to deliver the excitation laser in free space and collect the microlasing emission. In fact, the embedded NRs on the surface of WGMR act as the nanocouplers due to the Purcell effect and ensure coupling of a free space pulsed excitation laser.^102^ Therefore, an *in vivo* WGM microlaser can be realized upon what has been previously achieved. However, the main challenge would be the collection of microlasing and detection of the beatnote. This issue can be resolved by improving the quality and efficiency of the microlaser.

## Conclusion

Optoplasmonic WGM sensing provides a promising avenue for exploring biomolecular sensing. These sensors offer high sensitivity and the ability to detect molecular transitions such as conformational changes in enzymes. As a result, researchers now have new opportunities to investigate the kinetics and thermodynamics of enzymes. Additionally, these sensors can act as force gauges for femtonewton optical forces that affect conformational changes. By quantifying the thermodynamic free energy penalties applied during conformational transitions, researchers can gain insights into the underlying processes. Combining high precision, real-time single-molecule sensing, optical manipulation, and rapid feedback loops can enable the accurate on and off switching of enzymes. This opens new possibilities for research into error-free de novo DNA synthesis, which is important for biomanufacturing. Moreover, the combination of membrane-based single-molecule sensing with synthetic cell technology, which enables the expression of membrane proteins, could become a powerful platform for investigating static and dynamic membrane processes as well as protein and small molecule interactions with membranes. Thermo-optoplasmonic (TOP) sensing allows optoplasmonic WGM to be used as single-molecule absorption spectrometers, which is useful when determining the absorption cross-section on plasmonic nanosystems that exhibit unusual optical excitations and overcome forbidden transitions of molecules. This provides insights into the physicochemical molecular optics in a regime where the length scale of the electromagnetic field approaches that of the molecules, their bonds, and atoms. Finally, optoplasmonic WGM sensors have exciting prospects as active sensors and microlasers. By employing beat notes between counterpropagating WGM modes, researchers can achieve high detection sensitivities and reduce background noise to detect single-molecule events. In fact, active WGM sensors are currently being developed for *in vivo* sensing applications. Sensing enhancement can also be achieved using the physics of non-Hermitian active and passive systems and exceptional points by strategically placing NP scatterers on the microsphere. It can be seen that optoplasmonic WGM sensing offers exciting prospects for researchers, and we hope that this Perspective on the topic will stimulate new research directions in this field and allied sensor technologies.
